# Second line molecular diagnosis for bovine tuberculosis to improve diagnostic schemes

**DOI:** 10.1371/journal.pone.0207614

**Published:** 2018-11-26

**Authors:** Lorraine Michelet, Krystel de Cruz, Claudine Karoui, Jennifer Tambosco, Jean-Louis Moyen, Sylvie Hénault, María Laura Boschiroli

**Affiliations:** 1 University Paris-Est, French Agency for Food, Environmental and Occupational Health and Safety (Anses), Laboratory for Animal Health, Maisons-Alfort, France; 2 Laboratoire Départemental d'Analyse et de Recherche de Dordogne, Coulounieix Chamiers, France; Colorado State University, UNITED STATES

## Abstract

Surveillance of bovine tuberculosis (bTB) is partly based on the sanitary inspection of carcasses at the abattoir to detect bTB-like lesions which, in compliance with EU recommendations, are analysed by bacteriology and histopathology to disclose *Mycobacterium bovis* (or *M*. *caprae*) infection. Moreover, since 2012, a PCR method with similar sensitivity and specificity values of histopathology and bacteriology respectively is additionally employed in France, partially compensating for the weaknesses of classical diagnostic methods. We analysed a collection of bTB-like lesions from cattle presenting positive histological results albeit with negative PCR results. We present here the results of these samples, recovered from 292 animals culled between 2013 and 2016, analysed with a second line molecular diagnosis approach that consists in a combination of PCRs targeting the *M*. *tuberculosis*-*M*. *avium* complexes as well as the *Mycobacterium* genus and sequencing of *hsp*65 gene. These molecular analyses disclosed to identify the presence of non-tuberculous bacteria which could be responsible for most of these non-specific TB lesions: non tuberculous mycobacteria (24%) or Actinomycetales (56%) such as *Rhodococcus equi* (53%); 24% of the samples were negative. *M*. *bovis* -or any other MTBC members- was neither detected by molecular methods nor isolated in any of them at the end of the 3 months of culture. In conclusion, these results highlight the lack of specificity of histopathology and the usefulness of a first line PCR with a second line molecular diagnostic test to circumvent it. This diagnostic strategy makes it possible to reduce the number of suspect bTB cases raised at the abattoir or shortening their lock-up periods. By simplifying diagnostic schemes, the use of this tool could improve bTB surveillance and make eradication programs more efficient in the future.

## Introduction

Bovine tuberculosis (bTB), mainly due to *Mycobacterium bovis*, is an important re-emergent zoonotic disease in Europe [1]. Although France has been officially bTB free (OTF) since 2001, the persistence of the disease in livestock and its occurrence in wildlife in some areas is of great concern [2, 3]. The bTB control campaign in France, in compliance with the EU Directive 64/432, is mainly based on regular skin testing of animals and on detection of bTB-lesions during routine veterinary inspections at the abattoir. Indeed, inspection at the abattoir is a cost-effective method, especially in low prevalence areas or OTF countries [4]. In this particular context, the submission of all suspect lesions detected during meat inspection to the laboratory for histopathology and/or culture examination is necessary to increase the sensitivity of the surveillance system [5, 6]. Still, only by implementing more epidemiologically adapted control measures could eradication be envisaged [7].

BTB diagnosis by bacteriology can take up to three months due to the slow growth of the *Mycobacterium tuberculosis* complex (MTBC) mycobacteria [8]. Alternative more rapid tools such as histopathology are employed to circumvent the bacteriology slowness drawback. However, although histopathology is a fast and sensitive method it lacks specificity [9]. Actually, in a recent study on the evaluation of sensitivity and specificity of confirmatory bTB diagnostic tests, it was shown that histopathology was less specific than bacteriology, albeit as sensitive as another rapid test, a MTBC PCR introduced in France as a first line method, in parallel with bacteriology, to detect bTB infected animals [10]. This PCR method thus compensates for the specificity deficiency of histopathology which has been until now the only recognised rapid test in accordance with the EU Directive 64/432. Furthermore, the bovine tuberculosis National Reference Laboratory (NRL) applies a second line molecular diagnosis method that enables the identification of mycobacterial species either on mycobacteria bacteriological isolates or directly on DNA extracted from animal samples. This method provides rapid information about bTB or any other mycobacterial infection.

A significant and increasing number of cattle samples were analysed at the NRL, after discordant histopathological positive–first line PCR negative results. In this study we summarise the results of 4 years analyses on such samples with our second line molecular diagnosis scheme, which made it possible to identify non-tuberculous bacterial agents giving rise to nonspecific bTB-like lesions, to avoid cumbersome -albeit official- diagnostic alternatives such as culture for confirming the bTB-free status of the herd, thus gaining diagnostic specificity and confidence for bTB status confirmation.

## Materials and methods

### Ethical statement

BTB is a notifiable disease for which there are control and surveillance campaigns in France. Official methods for diagnosis of this disease are culture, PCR and histopathology. Therefore, all the samples included in this study are issued from animals analysed within an official context. No purpose killing of animals was performed for this study. All samplings were in complete agreement with national and European regulations. No ethical approval was necessary.

### Sample collection

Samples included in our study presented macroscopic bTB-like lesions at routine abattoir inspection between 2013 and 2016. Inspection procedures for bovine carcasses implemented in France (DGAL/SDSPA/SDSSA/N2013-8123, https://info.agriculture.gouv.fr/gedei/site/bo-agri/instruction-N2013-8123) follow the regulation(EC) No 854/2004 (Annex 1, Section IV, Chapter I). Typically, lesions due to *M*. *bovis* have a centre of caseous necrosis, sometimes associated with calcification, surrounded by epithelioid cells, lymphocytes and neutrophils [6, 11]. Samples had previously been submitted to first-line bTB diagnosis (bacteriology/PCR and histopathology) by authorised regional laboratories (RL) of the national surveillance network for bTB [10]. Briefly, histopathology was based on Hematoxylin-Eosine and Ziehl Neelsen staining. Bacterial culture is performed following the protocol established by the French NRL (NF U 47–104) for isolation of *M*. *bovis*. Two to 5 g of sampled tissues were crushed with a 4% sulfuric acid solution to decontaminate the tissue. After 10 min, the acid was neutralized by adding a 6% sodium hydroxide solution. After decontamination, the supernatant was seeded on two different solid media: Löwenstein-Jensen and Coletsos. All seeded media were incubated at 37°C +/- 3°C for three months and exanimated every two weeks. Any isolated mycobacterial strain is submitted to the NRL for further characterisation. DNA from each sample was extracted by using the QIAamp DNA mini kit (Qiagen, Courtaboeuf, France) or by Magvet MV384 (Thermo Fisher scientific, Villebon-sur-Yvette, France) with a King Fisher KF96 automate, following the manufacturer’s instructions and analysed with the LSI VetMAX *Mycobacterium tuberculosis* Complex Real-Time PCR Kit (Thermo Fisher scientific, Villebon-sur-Yvette, France). Samples submitted to the NRL for further molecular analyses were those that (i) presented a histopathological result suggesting tuberculosis, and (ii) showed a negative result with the first-line PCR.

Samples from 81 TB-free cattle without TB-like lesions following diagnostic slaughter and having presented a negative PCR result at the first-line bTB diagnosis were also included as a control population.

### Confirmatory tests-second line molecular diagnosis

For further analyses at the NRL, original tissue, macerated tissue and extracted DNA were sent by the RLs. A first analysis on DNAs was done by real-time PCR targeting insertion sequences IS*6110* and IS*1081* for MTBC identification, IS*1245* for *Mycobacterium avium* complex (MAC) identification, and the 65 kDa heat shock protein gene (*hsp65*) for *Mycobacterium* sp. detection (Table 1). Real-time PCR assays were performed in a final volume of 25 μl using the TaqMan Fast Universal PCR Master Mix (Roche Diagnostics, Meylan, France) at a 1X final concentration, with primers at 300 nM and probes at 250 nM. PCR cycling comprised of 2 min at 50°C and 20 s at 95°C, followed by 50 cycles of 2-step amplification of 3 s at 95°C, and 30 s at 60°C. If necessary (negative or doubtful results) a second analysis was done with a new DNA extraction with the High Pure PCR Template Preparation Kit (Roche Diagnostics, Meylan, France) from the original tissue and the macerated lesion. Quality of the DNA extraction and PCR inhibition was tested with DiaControlDNATM (Diagenode, Thermo Fisher, USAdiagnostics, Belgium).

**Table 1:** Primers and probes oligonucleotides for real-time-PCR assays used in this study.

Targeted genes or sequences	Primers-Probe name	Sequence 5’– 3’
IS*6110*	TR IS*6110* F	GGT AGC AGA CCT CAC CTA TGT GT
	TR IS*6110* R	AGG CGT CGG TGA CAA AGG
	TR IS*6110* P	(FAM)-CAC GTA GGC GAA CCC-(MGB-NFQ)
IS*1081*	TR IS*1081* F	CCG CCA CCG TGA TTT CGA
	TR IS*1081* R	GCC AGT CCG GGA AAT AGC T
	TR IS*1081* P	(FAM)-CCG CAA CCA TCG ACG TC-(MGB-NFQ)
IS*1245*	TR IS*1245* F	GCC GCC GAA ACG ATC TAC
	TR IS*1245* R	TGA CCC GGT GCG CAG CTT
	TR IS*1245* P	(FAM)-TCG CGT CCG CGC ACG CTG TCC A-(BHQ1)
Hsp65	F MSP	GCC AAG GAG GTC GAG ACC AA
	R MSP	CTC CTC GAC GGT GAT GAC
	P MSP	(FAM)-ACC TTG TCC ATC GCC TCG GCG AT-(BHQ1)

Identification of non-tuberculous mycobacteria species was done by sequencing using primers targeting the 65 kDa heat shock protein gene (*hsp65*) [12] or the β subunit of bacterial RNA polymerase sequence (*rpoB*) [13]. The obtained sequences were compared to the GenBank/EMBL/DDBJ databases using the BLAST program.

## Results

This retrospective analysis includes all cattle cases sent to the NRL which had a negative result for MTBC PCR but with a bTB suggestive histopathology. Between 2013 and 2016, we analysed samples of 292 cattle from 278 herds (Table 2). The majority (91%) of samples were draining lymph nodes (LN) (102 retropharyngeal, 67 mediastinal and 96 tracheobronchial), while the remaining samples were a few other LN (6%) and organs (liver or lung) (3%).

**Table 2:** Number of histology +/PCR—Samples analysed and final diagnosis (bacteriology or molecular) obtained at the National Reference Laboratory for Tuberculosis.

		Final diagnosis		
	Total	Non tuberculous mycobacteria	Actinomycetales	Negative
Number of bovine	292*	69	164	70
Number of corresponding herd	278	67	158	69
Retropharyngeal LN	102	26	65	16
Tracheobronchial LN	96	18	54	27
Mediastinal LN	67	14	39	15
Other LN	18	9	4	5
Organs	9	1	1	7

LN: Lymph node

* Eleven samples were co-infected by two bacteria

^♦^ Several LNs (1–5) per cattle were analysed

The histopathological profiles of these animals were almost the same, i.e. encapsulated granulomas with necrosis areas and the presence of Langhan’s giant cells. For some samples, other types of cells, like lymphocytes or macrophages, were observed as well as a partial to complete mineralisation. The identification of acid-alcohol resistant bacillus by Ziehl Neelsen staining was positive for a few samples (15/292), latter identified as MAC (n = 11) or *Mycobacterium* sp. (n = 1), *R*. *equi* (n = 1) and a *Nocardia* sp. (n = 1).

Only 31 out of the 292 samples were bacteriology positive. Identification of these isolates was performed by *hsp65* sequencing: 13 were MAC, 15 were other non-tuberculous mycobacteria (NTM) (1 *M*. *aichiense*, 2 *M*. *bourgelatii*, 2 *M*. *kansasii*, 8 *M*. *nonchromogenicum*, 1 *M*. *petroleophilum*, and 1 *M*. *pyrenivorans*) and four *Rhodococcus equi*. The identification by bacteriology was congruent with the one on tissue for 22 samples (S1 Table). For the other nine samples, the results suggest co-infection as different pathogens were identified by bacteriology and PCR on tissue samples.

Of the 292 cases submitted to the NRL, 24% were NTM, 56% were Actinomycetales and 24% were negative based on sequencing (Table 2). In 11 cases, we identified co-infection, either between two NTM (*M*. *avium avium* and *M*. *nonchromogenicum*) or between a NTM (*M*. *aichiense*, *M*. *bourgelatii*, *M*. *kansasii*, *M*. *nonchromogenicum* or MAC) and an Actinomycetale (*Rhodococcus equi*). Identification of the bacteria species by sequencing highlighted that 95% of the Actinomycetales were *R*. *equi*. Among the NTM, MAC represents 26% of the cases, while the other half were various mycobacteria species (Table 3). These NTM and Actinomycetales were found in various samples, most frequently in retropharyngeal LN (31%), followed by tracheobronchial LN (25%), mediastinal LN (18%), other LN (4%) or organs (0.7%). More than 55% of the retropharyngeal and tracheobronchial LN was infected by *R*. *equi*.

**Table 3:** Bacteria identified by *hsp65* and *rpoB* sequencing from DNA extracted from lymph nodes with bTB-like lesions.

Group	Species	N
Non tuberculous mycobacteria	*M*.* aichiense*	1
	*M*.* avium avium*	18
	*M*.* avium hominissuis*	4
	*M*.* avium paratuberculosis*	5
	*M*.* bourgelatii*	2
	*M*. *genavense*	1
	*M*.* gordonae*	1
	*M*.* intracellulare*	1
	*M*.* kansasii*	3
	*M*.* nonchromogenicum*	9
	*M*. *petroleophilum*	1
	*M*.* pyrenivorans*	1
	*M*.* shimoidei*	1
	*M*. *thermoresistibile*	1
	*Mycobacterium* sp.	20
*Actinomycetales*	*Gordonia* sp.	1
	*Nocardia* sp.	5
	*Rhodococcus erythropolis*	2
	*Rhodococcus equi*	155
	*Rhodococcus pyridinivorans*	1
**Total**		233*

*Eleven samples were co-infected.

Of the 240 LN from control animals without bTB-like lesions and a negative first-line PCR results, 96% remained completely negative to the second line diagnosis (Table 4). Only two NTM were identified in mediastinal LN (*M*. *lentiflavum* and *M*. *gordonae*) and 8 actinobacteria from various genus: *Corynebacterium* sp., *Streptomyces* sp., *Arthobacter* sp. and *Nakamurella* sp. These results confirm that bacteria identified in nonspecific bTB-like lesions are not ubiquitous and accordingly that they are the real causative agents of them.

**Table 4:** Number of histology -/PCR—Samples analysed and final diagnosis (bacteriology or molecular) obtained at the National Reference Laboratory for Tuberculosis.

		Final diagnosis		
	Total	Non tuberculous mycobacteria	Actinomycetales	Negative
Number of bovine	81^♦^	2	8	230
Number of corresponding herd	42	2	7	42
Retropharyngeal LN	72	0	1	71
Tracheobronchial LN	72	0	5	67
Mediastinal LN	71	2	2	67
Other LN	25	0	0	25

LN: Lymph node

* Eleven samples were co-infected by two bacteria

^♦^ Several LNs (1–5) per cattle were analysed

## Discussion

We studied bTB suspect cases resulting from abattoir inspection which presented nonspecific histopathology bTB-like lesions and a negative first line MTBC PCR and focused on the identification of the bacteria responsible for them. The number of this type of nonspecific suspicions increased since 2013 as a result of awareness campaigns organised for abattoir agents, one of a series of measures introduced to reinforce the national bTB control campaign in 2011 [14]. None of these cases were real bTB infections, but other bacteria, i.e. NTM or Actinomycetales, have been identified in the lesions. Indeed, bTB-like lesions could also be caused by other granuloma forming organisms such as NTM or *Nocardia* species [15]. Moreover, mycobacteria and some Actinomycetales (of the genus *Nocardia*, *Rhodococcus* or *Corynebacterium*) shared the same tinctorial properties and thus are identified as acid resistant bacilli by Ziehl Neelsen staining [16].

Many environmental mycobacteria may interfere with the bTB surveillance program at post-mortem inspections at the abattoir [17]. NTM have recently been detected in lymph nodes of clinically healthy Swiss cattle, emphasizing the need of more specific diagnostic tools [18]. A study in Northern Ireland tried to identify mycobacteria in lymph nodes of cattle belonging to herds with previous evidence of bTB. The identified bacteria species were almost the same as in our study but with different proportions: a majority of *M*. *nonchromogenicum*, few MAC, few *M*. *kansasii* and only one *R*. *equi* [19]. NTM as well as *R*. *equi*, have been recognised in lymph node infection of domestic and wild animals (swine and wild boar (*Sus scrofa*), roe deer (*Capreolus capreolus*) or red deer (*Cervus elaphus*)), which could lead to a possible misdiagnosis of *M*. *bovis* [20–22]. Indeed, real tuberculosis lesions in cattle are commonly found in retropharyngeal LN (29.4%), mediastinal LN (28.2%) and tracheobronchial LN (18%) [6], i.e. the same locations as the non-tuberculous agents in our study.

Our results strongly suggest the link between *R*. *equi* and the presence of nonspecific bTB-like lesion as this species was not identified in the LN of cattle without any bTB-like lesions. *Rhodococcus equi* and non-tuberculous mycobacteria (especially MAC) are facultative intracellular pathogens surviving inside macrophages and inducing granulomatous inflammation [17, 23]. *Rhodococcus equi* (formerly *Corynebacterium equi*) is a coccobacillus bacterium commonly found in soil which is pathogenic for domesticated animals such as horses, pigs and cattle [24, 25]. Even if its pathogenicity is low in cattle, it may occasionally cause lymph node granulomas, which are detected at abattoir post-mortem examination [26]. The interference caused by *R*. *equi* in the monitoring of bTB was already acknowledged 35 years ago [27]. This bacterium has the capacity to modify the phagocytic vacuole of host macrophages and present similarities on cellular responses attributed to resemblances in cell wall composition and antigenic structure with bTB agents [9, 25]. Granulomas caused by *R*. *equi* are most frequently observed in retropharyngeal, bronchial and mediastinal lymph nodes [25] and are really difficult to differentiate from *M*. *bovis* granulomas, even if the presence of a heavy infiltration of neutrophils and/or extensive sheets of macrophages could presumably allow distinction [26]. Non-tuberculous mycobacteria and MAC are ubiquitous in the environment and particularly found in wet soil, water or plants [20, 22]. Infection of animals in our study probably occurred by the oral route through ingestion of food or water contaminated by these environmental organisms [25]. Some of the identified NTM species are recognised as leading to misdiagnosis of bovine tuberculosis, especially MAC [17] and *Mycobacterium nonchromogenicum* which is known to interfere with ante-mortem diagnosis of bTB [17, 28, 29]. The role of the other identified species as cattle pathogens is unclear. *Mycobacterium bourgelatii*, closely related to *M*. *intermedium* and described for the first time in 2013, was isolated from cattle lymph nodes [30]. *M*. *intermedium* is classified as a ‘pathogen’, together with *M*. *gordonae* and *M*. *kansasii*, in a recent phylogenetic analysis [31]. Thus, their role in nonspecific bTB diagnosis cannot be ruled out [17]. *Mycobacterium aichiense* closely related to *M*. *gilvum* [32], *M*. *petroleophilum* closely related to *M*. *aurum* [33], *M*. *pyrenivorans* and *M*. *thermoresistibile* are all rapidly growing mycobacteria found in the environment, potentially opportunistic in humans and included in the same phylogenetic group [31]. *Mycobacterium genavense*, slow growing mycobacteria, is responsible for infection both in birds and humans and has also been isolated from the environment [34]. *Mycobacterium shimoidei*, slow growing mycobacteria, is an opportunistic pathogen of humans but few pulmonary cases have been reported worldwide [35].

In our study, the majority of the samples presented a histological profile with an encapsulation of the granuloma and the presence of giant Langhans cells, sometimes in association with others cells (lymphocytes, neutrophils or epithelioid cells). The presence of epithelioid macrophages and Langhans cells is not pathognomonic as these cells are seen in immunologic granulomas [36], especially in tuberculous ones [37]. The histopathological results of our cases were quite the same for lesions due to NTM or Actinomycetales, indicating a clear lack of specificity in histopathological diagnosis. However, the use of immunohistochemistry, in complement of special staining, could have increased the specificity of this diagnostic test by demonstrating *M*. *bovis* antigen immuno-localisation [36]. In a previous study, the sensitivity and specificity of confirmatory tests (bacteriology, histopathology and PCR) were estimated under French field conditions [10]. Histopathology was found to be as sensitive as PCR but less specific than bacteriology or PCR, which means that this test cannot be used alone as a confirmatory test. Our results confirm and explain histopathology’s lack of specificity. Besides, second line PCR showed its usefulness as it clearly improves bTB diagnosis by disclosing false MTBC-infected cases. The second line molecular tests used at the NRL have shown an excellent negative predictive value and have quickly reduced the number of bTB suspicions through the identification of other non-tuberculous bacteria; however, as the proposed molecular diagnosis scheme is not a EU officially recognised strategy, animal movement restrictions in the 278 incriminated herds were maintained during at least 3 months awaiting for declaration of a negative *M*. *bovis* culture. In conclusion, second line molecular tests used at the NRL could confidently be added as an official test in order to accelerate the diagnosis process and improve bTB surveillance in Europe and to render control and eradication programs more efficient in the future.

## Supporting information

S1 Table Congruence of culture and PCR results. (DOCX)
