# Supplementary material for: Second line molecular diagnosis for bovine tuberculosis to improve diagnostic schemes
Source: PLoS One. 2018 Nov 26;13(11):e0207614. doi: 10.1371/journal.pone.0207614 (PMC6261039; doi:10.1371/journal.pone.0207614)
Supplement: S1 Table — (DOCX) [file pone.0207614.s001.docx]

**Supporting information**

**S1 Table. Congruence of culture and PCR results**

|  | Bacteriology | PCR on tissue | N |
| --- | --- | --- | --- |
| Concordant results (N=22) | MAC | | 12 |
|  | *R. equi* | | 4 |
|  | *M. nonchromogenicum* | | 3 |
|  | *M. bourgelatii* | | 1 |
|  | *M. kansasii* | | 1 |
|  | *M. pyrenivorans* | | 1 |
| Non concordant results (N=9) | *M. nonchromogenicum* | *R. equi* | 4 |
|  | *M. aichiense* | *R. equi* | 1 |
|  | *M. bourgelatii* | *R. equi* | 1 |
|  | *M. kansasii* | *R. equi* | 1 |
|  | MAC + *M. nonchromogenicum* | MAC | 1 |
|  | *M. petroleophilum* | *Mycobacterium* sp. | 1 |
